# Differential Analysis of A-to-I mRNA Edited Sites in Parkinson’s Disease

**DOI:** 10.3390/genes13010014

**Published:** 2021-12-22

**Authors:** Denis V. Pozdyshev, Anastasia A. Zharikova, Maria V. Medvedeva, Vladimir I. Muronetz

**Affiliations:** 1 Belozersky Institute of Physico-Chemical Biology, Lomonosov Moscow State University, 119234 Moscow, Russia; vimuronets@belozersky.msu.ru; 2 Faculty of Bioengineering and Bioinformatics, Lomonosov Moscow State University, 119234 Moscow, Russia; azharikova89@gmail.com (A.A.Z.); maryshick@mail.ru (M.V.M.)

**Keywords:** post-transcriptional RNA modification, A-to-I editing, Parkinson’s disease, RNA-Seq, REDItools, REDIportal

## Abstract

Parkinson’s disease (PD) is a widespread neuronal degenerative disorder with unexplored etiology. It is associated with various pathological events. In particular, the prefrontal cortex Brodmann area 9 (BA9) region is affected in PD. This frontal lobe brain region plays an important role in cognitive, motor, and memory-related functions. BA9 develops Lewy bodies in PD patients and shows essential changes in transcriptome and proteome, connected with mitochondria related pathways, protein folding pathways, and metallothioneins. Recently, altered adenosine to inosine mRNA editing patterns have been detected in various neurological pathologies. In this article, we present an investigation of differences in A-to-I RNA editing levels and specificity of mRNA editing sites in brain tissues of healthy and PD patients based on RNA sequencing data. Overall, decreased editing levels in the brains of PD patients were observed, potential editing sites with altered editing during PD were identified, and the role of different adenosine deaminases in this process was analyzed.

## 1. Introduction

Parkinson’s disease (PD) is the most common age-related motoric neurodegenerative disease that greatly affects one’s overall quality of life. The etiopathogenic mechanisms that underlie this illness are not yet fully understood. PD is considered to be a disease of the neuronal system characterized by the depletion of dopamine neurons in substantia nigra pars compacta [1] and the buildup of Lewy bodies (LBs) comprised of misfolded proteins [2]. The most prevalent component of LBs is α-synuclein and mutations in this protein are associated with a familial form of PD [3]. 

Besides the dopaminergic nigrostriatal system, PD also affects the frontal and prefrontal cortex [4,5]. Thus, reduced neuronal activity in Brodmann area 9 brain region (BA9) [6] and LBs formation in the prefrontal cortex were observed in PD patients [7].

To date, a large number of studies have shown that adenosine to inosine (A-to-I) mRNA editing is involved in neurological and neurodegenerative diseases in humans [8]. A-to-I editing is the post- or co-transcriptional modification of mRNA nucleotides potentially influencing RNA structure and function. The brain is one of the organs preferentially targeted by RNA editing enzymes. Amyotrophic lateral sclerosis [9], Alzheimer’s disease, Huntington’s disease [10], and many neurological developmental disorders are among disorders associated with changes in A-to-I RNA editing patterns. Thus, the human brain seems to be highly vulnerable to dysregulation of RNA editing, as above-mentioned. However, PD is yet to be investigated for RNA editing levels and specificity. Therefore, a search for potential edited sites in the BA9 brain region and differential analysis of such sites was conducted to fill this gap. The analysis was performed on RNA-Seq data from open sources [11]. 

## 2. Materials and Methods

### 2.1. Data

Raw sequencing reads of post-mortem prefrontal cortex (BA9) samples from 29 individuals with PD and 44 neurologically healthy individuals were analyzed [11]. All control and PD brain samples were derived from males of European ancestry without significant Alzheimer’s disease pathology. The average age of death for the PD group was 77.55 and 70 years for the control group. Original data are freely available from the Sequence Read Archive (SRA) (https://www.ncbi.nlm.nih.gov/bioproject/PRJNA283498, accessed on 7 November 2020). For RNA extraction, brain samples were homogenized in TRIzol (Invitrogen, Carlsbad, CA, USA), then total RNA fraction was isolated using the Qiagen RNeasy Mini Kit (Qiagen Sciences Inc., Germantown, MD, USA) and purified using Agencourt RNA clean magnetic beads (Beckman Coulter, Inc., Carlsbad, CA, USA). RNA-Seq library preparations were made using Illumina’s TruSeq RNA Sample Prep Kit according to the manufacturer’s protocol. Samples were sequenced using 2 × 100 nt paired-end runs on Illumina’s HiSeq 2000 system [11]. 

### 2.2. Bioinformatic Tools

The quality assessment of the sequences was performed using FastQC software v0.11.8 (http://www.bioinformatics.babraham.ac.uk/projects/fastqc/, accessed on 7 November 2020). 

Paired-end reads were trimmed from the 3′ end to remove low-quality bases (TRAILING:20) to a minimum length of 25 nt using Trimmomatic v. 0.39 [12]. Trimmed paired-end reads were mapped on the human reference genome (hg38) with the program hisat2 (version 2.0.5). 

The REDItools (v 1.2.1) software was used (REDItoolDenovo.py) to identify potential RNA editing sites in RNA-Seq data. We tuned a list of REDItools parameters to identify more potential editing sites, multi-hits and duplicates from reads alignment were excluded (parameters -e and -d, respectively), and only reads in concordant pairs were used (parameter -p). The filter for mapping the quality score was set to 60 (parameter -m) according to REDItools recommendations for the Hisat2 aligner and the minimum quality score was set to 30 (parameter -q). Minimum read coverage and the minimum number of reads supporting the variation were defined as one read (parameters -c and -v, respectively) (GitHub—BioinfoUNIBA/REDItools: REDItools are python scripts to investigate RNA editing at genomic scale.). 

Differential expression analysis was performed using DESeq2 [13] with BH-adjusted *p*-values. 

### 2.3. Statistical Analysis

The Fisher’s test with BH-adjusted *p*-values was used to compare the frequencies of potential editing sites between the control and PD groups. 

Wilcoxon test was used to reveal the difference in the number of potential A-to-I mRNA editing sites between groups of PD and neurologically healthy people.

## 3. Results

mRNA editing is a common phenomenon in mammals, but its functional role is still largely unknown. The most prevalent type of mRNA editing is the conversion of adenosine to inosine (A-to-I editing) made by adenosine deaminases. These proteins are composed of the ADAR protein family including ADAR1 and ADAR2, which are catalytically active [14], and ADAR3, which is thought to be catalytically inactive [15]. ADAR proteins are involved in mechanisms of alternative splicing regulation and transcriptional control. Modifications in protein-coding regions can lead to amino acid replacements with subsequent functional changes [16]. The rapid development of next-generation sequencing technologies has accelerated the discovery of new RNA editing sites, and so far, more than 15 million modification sites have been identified in humans [17]. It is important to mention that only a small part of RNA editing events occurs in protein-coding regions and leads to non-synonymous amino acid substitutions. Such substitutions are primarily observed in neural tissues and are over-represented in transcripts of genes linked to the nervous system function [18]. 

**Figure 1:**
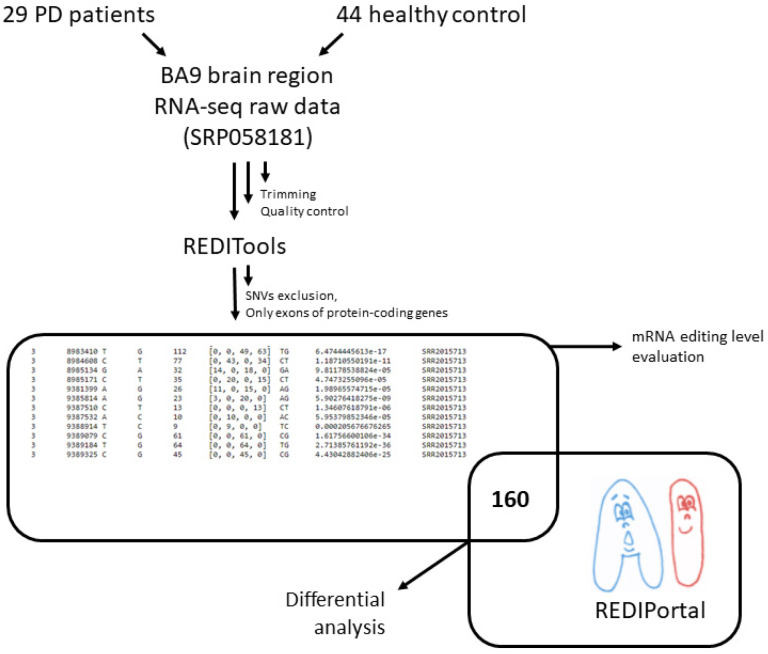
The workflow of data preprocessing, differential RNA edited site analysis, and RNA editing level evaluation.

Several methods allow for the identification of RNA editing sites in transcriptome datasets [19]. Some have been developed to repurpose RNA-Seq data for the RNA editome investigation. In this study, we used the REDItools software package feature to identify RNA editing sites in the absence of matched genomic DNA sequences (Figure 1, [20]).

A set of the prefrontal cortex (BA9) samples from PD patients and neurologically healthy controls (sex and age-similar) were analyzed [11]. Raw reads of these samples (SRP058181) are freely available at the Sequence Read Archive (SRA) (https://www.ncbi.nlm.nih.gov/bioproject/PRJNA283498, accessed on 7 November 2020). The analyzed RNA-Seq data included on average 42,166,654 million raw paired-end reads (100 bases × 2) per sample and had been generated by the Illumina HiSeq 2000. Trimmed paired-end reads were mapped on the human reference genome (hg38) with the program hisat2 (version 2.0.5). The BAM alignments were processed with the REDItoolDenovo.py script included in the REDItools package. The script provided the list of potentially edited positions as a textual table containing coverage depth, the mean quality score, the observed base distribution, the strand, the list of observed substitutions, and the variation frequency. Only hits within gene protein-coding regions were taken into account. 

The obtained dataset of potential RNA editing sites was additionally filtered: sites described earlier as potential single nucleotide variations from the gnomAD database (https://gnomad.broadinstitute.org/, accessed on 9 April 2019) were excluded, and those matched to known RNA editing sites from the REDIportal were taken for further analysis. There were 160 records in the final table. The number of samples with edited bases was counted in the healthy control and PD patient samples for each position. These numbers were compared using Fisher’s test and Bonferroni multiple testing correction to identify sites showing statistically significant differential editing.

Therefore, sites with differential mRNA editing in PD were identified (Table 1). Notably, half of the determined positions belong to genes encoding glutamate ionotropic receptors, which play key roles in synaptic plasticity [21]. One other ionotropic receptor GABA_A_ reduces neuronal excitability by inhibiting nerve transmission [22]. Abnormalities in mRNA editing of these genes could be associated with non-motor symptoms of PD such as anxiety or problems with memory. *GIPC1* was recently identified as a gene likely to be involved in the development of PD [23]. The role of perilipin Plin4 (a coating protein and regulator of intracellular lipid droplets) in neuronal cytotoxicity has also been shown in PD experimental models [24]. Thus, altered mRNA editing profiles of identified genes could be part of the PD pathogenesis mechanism.

**Figure 2:**
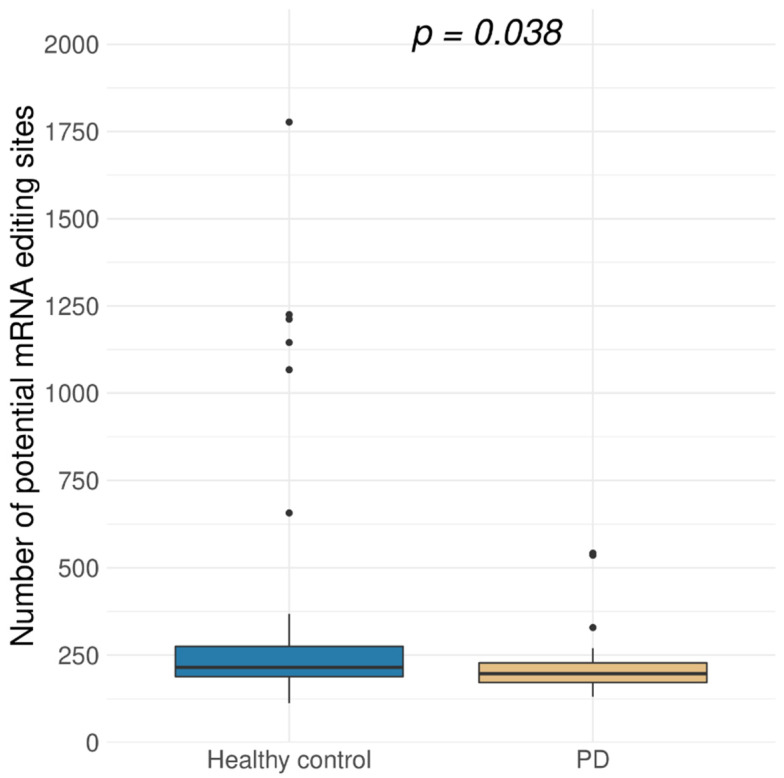
Distribution of the number of potentially edited A-to-I sites in exonic regions of protein-coding genes in brain samples for Parkinson’s disease (PD, 29 samples) and healthy control (44 samples) (*p* = 0.038, Wilcoxon test). Potentially edited sites were predicted using REDItools software [20].

Among 160 potentially edited positions, found both in brain RNA-Seq data and the REDIPortal database, most positions were more frequently edited in neurologically healthy people. This notice led to a hypothesis that the overall level of A-to-I mRNA editing in PD was reduced. To check this hypothesis, an extended set of positions was analyzed (Figure 1). Namely, found sites were filtered as previously described, except all hits matched and unmatched to known RNA editing sites from the REDIportal were taken for further analysis. Presence of rare SNVs in the remaining pool of RNA variants is possible, but the same editing sites are often present in different individuals, whereas rare SNVs are most likely not, so we suppose that this did not affect the statistical test results. The level of A-to-I editing was significantly lower for protein-coding transcriptome in PD samples than in the healthy controls (Figure 2). We noticed that the number of discovered potentially edited sites correlated with sequencing library size, but the normalized data also showed that the control samples were edited more frequently. 

A-to-I substitutions are mediated by the adenosine deaminase family of enzymes that act on RNA (ADAR). Three ADAR genes have been identified in humans, and only two of them encode enzymes with mRNA editing activity (*ADAR* and *ADARB1*). The differential expression analysis was performed using DESeq2 to determine relative mRNA expression of these genes (Table 2). 

*ADAR* gene expression was significantly lower in the PD patients’ samples (*p*-value < 0.1). This statement correlates with lower levels of A-to-I editing in PD patients. Therefore, *ADAR* gene expression aberrations could be included in the pathogenesis of this neurodegenerative disease.

## 4. Discussion

Recently, RNA editing has been added to the list of common post-transcriptional modifications. It has been shown that ADAR enzymes could change A-to-I nucleotides in RNAs, affecting gene expression. Most identified editing sites reside within repeated sequences and introns [25]. Such editing is considered to induce changes in pre-mRNA splicing by altering splice site recognition sequences. Translation machinery recognizes inosine in protein-coding regions of RNA as guanosine, which may lead to amino acid substitutions. Converted A-to-I positions leading to non-synonymous amino acid substitutions (so-called “recoding positions”) represent a much smaller group of known mRNA edited sites. 

A-to-I RNA editing in coding regions is quite rare, but many are associated with neuronal functions. Altered editing levels in specific coding sites have been reported for several neurological and neurodegenerative disorders such as major depression, epilepsy, schizophrenia, amyotrophic lateral sclerosis (ALS), and Alzheimer’s disease (AD). In most mentioned cases, A-to-I conversion is decreased due to ADAR downregulation [8]. Generally, RNA editing connection to central nervous system pathogenesis comes down to a small fraction of recoding sites. For ALS, it is the Q/R site in GluA2—a subunit of AMPA type ligand-gated ion channel that mediates an influx of extracellular Na^+^ and/or Ca^2+^, regulating membrane depolarization. It is considered that decreased editing leads to exaggerated Ca^2+^ influx through that glutamate receptor and subsequent lower motor neuron death [26]. In brain samples of Huntington’s (HD) and AD patients, the decreased editing of the Q/R site in GluA2 also takes place [10,27]. It has been shown that during AD, editing levels decreased mainly in the hippocampus and to a lesser degree in the temporal and frontal lobes. Altered RNA editing levels for AD patients were observed in 22 genes. The participation of RNA-edited genes in the pathogenesis of HD and AD has not been fully characterized yet [28]. 

This work presents data on changes in the editing level for exons of protein-coding genes in PD. For patients with an established diagnosis, a decrease in the level of editing along with significantly lower expression of *ADAR* and *ADARB1* genes have been observed. These data are in accordance with previously reported evaluations of RNA editing levels in other neurodegenerative diseases. Increased expression of *ADARB2* in PD samples (Table 2) could also contribute to general editing level decrease as ADAR3 has been shown to be a competitive inhibitor of ADAR1 and ADAR2 in vitro [15].

Specific substitution sites associated with the PD have also been identified. Edited sites have been found among the mRNA of genes of the subunits of the kainite and AMPA types in ionotropic glutamate receptors; it has previously been shown that ionotropic glutamate receptor antagonists could have antiparkinsonian action [29]. Thus, altered mRNA editing of these genes may result from compensatory mechanisms in PD. Notably, a Y571C substitution in the GRIK2 protein is a potentially dysregulated RNA editing event in HD [20]. One more differentially edited site in *GABRA3* (I342M), detected in this work, has previously been shown to be under-edited in lower grade glioma and glioblastoma [30]. As the main inhibitory neurotransmitter within the nervous system, γ-aminobutyric acid (GABA) is involved in a wide variety of physiological functions that are maintained through a complex interaction between GABA and calcium-dependent neurotransmission [31]. Therefore, finding the GABRA3 subunit of the GABA receptor among the edited genes may also be important. Two other genes, *PLIN1* and *GIPTC1*, have recently been shown to be associated with PD. Although the obtained data need experimental verification, the first evidence for A-to-I editing in the pathogenesis of this neurodegenerative disease has been obtained.

## 5. Conclusions

An approach for selecting candidate A-to-I editing sites involved in PD disease was presented. This approach is based on the repurposing of open RNA-Seq data without matching genomic sequences using REDItools features. PD-specific sites with altered editing were determined. Observed lower levels of ADAR gene expression and overall decreased editing levels in PD samples could testify that mRNA modification patterns act as a part of the pathogenesis of this neurodegenerative disease.

## Figures and Tables

**Table 1:** Genes with differentially mRNA edited sites within protein-coding regions in PD patients’ brains versus the healthy control.

Gene	Chromosome	Coordinate	Edited in Control	Edited in PD	Fisher Test	p.adj.
*GIPC1*	chr19	14482881	38/44	11/29	3.00 × 10^−5^	4.80 × 10^−3^
*GRIA2*	chr4	157336727	30/44	6/29	1.00 × 10^−4^	8.00 × 10^−3^
*PLIN4*	chr19	4511513	9/44	18/29	4.80 × 10^−4^	2.56 × 10^−2^
*GABRA3*	chrX	152189847	37/44	14/29	1.67 × 10^−3^	6.68 × 10^−2^
*GRIK2*	chr6	101889827	27/44	7/29	2.10 × 10^−3^	6.72 × 10^−2^
*GRIK1*	chr21	29581430	30/44	9/29	3.65 × 10^−3^	9.73 × 10^−2^

**Table 2:** Differential expression analysis of RNA-specific adenosine deaminases in PD patients’ brains versus the healthy control.

Gene	log2FoldChange	p.adj.
*ADAR*	−0.150973114	0.06
*ADARB1*	−0.296571997	0.10
*ADARB2*	0.341071508	0.28

## Data Availability

Sequence Read Archive (SRA) (https://www.ncbi.nlm.nih.gov/bioproject/PRJNA283498, accessed on 7 November 2020).
